# 2261 Tumor suppressor RARRES1 regulates cell survival by modulating mitochondrial energetics

**DOI:** 10.1017/cts.2018.142

**Published:** 2018-11-21

**Authors:** Sara Maimouni, Mi-Hye Lee, You-Me Sung, Chokri Ouaari, Stephen Byers

**Affiliations:** 1 Georgetown University – Howard Universities; 2 Lombardi Comprehensive Cancer Center; 3 University of the District of Columbia

## Abstract

OBJECTIVES/SPECIFIC AIMS: One of the driving mechanisms of cancer progression is the reprogramming of metabolic pathways in intermediary metabolism. Cancers increase their energy expenditure by increasing ATP production for utilization in anabolic pathways to increase production of proteins, nucleic acids and lipids. The Warburg effect, where cancer cells predominantly use aerobic glycolysis rather than oxidative phosphorylation to produce ATP, was long thought to be the main initiating pathway in increasing tumor burden. However, compelling new evidence shows that there exists metabolic heterogeneity among and within tumors. Mitochondrial respiration often plays a major role in tumor progression, as many different cancers contain a subpopulation of slow-cycling tumor-initiating cells that are multidrug-resistant and dependent on oxidative phosphorylation. These cells represent a target for cancer therapy. In this study, we identification a novel endogenous regulator of mitochondrial respiration, retinoic acid receptor responder 1 (RARRES1). METHODS/STUDY POPULATION: We assessed the metabolic phenotype of RARRES1-depleted normal epithelial cells through metabolomics, a flux analyzer and blotting for phosphorylation of AMP kinase, a major regulator of energy homeostasis. We further examined mitochondrial energetics by staining the mitochondria with TMRM and Mito-Tracker. We then analyzed the apoptotic phenotype of epithelial cells with depletion of RARRES1 with fluorescence-activated cell sorting analysis of annexin V-staining. RESULTS/ANTICIPATED RESULTS: Remarkably, fluorescence-activated cell sorting analysis of annexin V-stained epithelial cells with depletion of RARRES1 were resistant to all studied modes of cell death, implying an effect on a fundamental cell process. By using proteomics, metabolomics, cellular and molecular analyses, our data show that RARRES1 regulates mitochondrial membrane potential and subsequently alters 1-carbon metabolism by modulating the function of the mitochondrial voltage-dependent anion channel. We believe this is the first example of a tumor suppressor protein that functions to directly regulate mitochondrial energetics. Using an extracellular flux analyzer, our data also show that depletion of RARRES1 causes an increase in mitochondrial respiration and ATP production, thus enhancing biosynthetic pathways that drive the pathogenicity and survival of cancer. The metabolic and anti-apoptotic phenotype of RARRES1-depleted cells was reversed by treatment of metformin, a mitochondrial inhibitor. DISCUSSION/SIGNIFICANCE OF IMPACT: These data lay the foundation for metabo-therapy of the many tumor types that exhibit RARRES1 depletion and may have the added benefit of targeting drug-resistant tumor-initiating cells.

